# 

**Published:** 1883-02

**Authors:** 


THIRTIETH YEAR.

Mar 19 0

### HALL'S JOURNAL OF HEALTH

Guaranteed Circulation 200 000 Copies in 12 Months.

*E. H. GIBBS, A.M. M.D., Editor,*

No. 135 EIGHTH STREET (Near Broadway), NEW YORK.

TERMS: $2 a Year, or $1 for Six Months. Single number, 20 cts.

